# Neuropsychiatric Disorders: Influence of Gut Microbe to Brain Signalling

**DOI:** 10.3390/diseases6030078

**Published:** 2018-09-06

**Authors:** Mary Scriven, Timothy G. Dinan, John F. Cryan, Mary Wall

**Affiliations:** 1 Department of Psychiatry, University College Cork, T12 DC4A Cork, Ireland; mary.scriven@hse.ie (M.S.); Mary.Wall5@hse.ie (M.W.); 2 APC Microbiome Ireland, University College Cork, T12 YT20 Cork, Ireland; j.cryan@ucc.ie; 3 Department of Anatomy and Neuroscience, University College Cork, T12 XF62 Cork, Ireland

**Keywords:** psychiatry, gut microbiome, probiotics

## Abstract

The microbiome gut brain (MGB) axis consists of bidirectional routes of communication between the gut and the brain. It has emerged as a potential therapeutic target for multiple medical specialties including psychiatry. Significant numbers of preclinical trials have taken place with some transitioning to clinical studies in more recent years. Some positive results have been reported secondary to probiotic administration in both healthy populations and specific patient groups. This review aims to summarise the current understanding of the MGB axis and the preclinical and clinical findings relevant to psychiatry. Significant differences have been identified between the microbiome of patients with a diagnosis of depressive disorder and healthy controls. Similar findings have occurred in patients diagnosed with bipolar affective disorder and irritable bowel syndrome. A probiotic containing *Lactobacillus acidophilus*, *Lactobacillus casei*, and *Bifidobacterium bifidum* produced a clinically measurable symptom improvement in patients with depressive disorder. To date, some promising results have suggested that probiotics could play a role in the treatment of stress-related psychiatric disease. However, more well-controlled clinical trials are required to determine which clinical conditions are likely to benefit most significantly from this novel approach.

## 1. Introduction

Psychiatric illness is estimated to account for over 32 percent of years lived with disability and 13 percent of disability-adjusted life years [1]. Unfortunately, despite the multiple pharmacological and psychological treatment options available, many patients with psychiatric diagnoses remain disabled by their symptoms. Additionally, some of the side-effects produced by psychotropic medication impact negatively on patient’s functioning, examples include weight gain and sexual dysfunction [2]. Simultaneously, probiotics have become widely available with their multiple claims (often exaggerated) being advertised to the general public through various media channels. It is, therefore, not surprising that the use of probiotics to improve medical and psychiatric symptoms has garnered much excitement and anticipation. The use of probiotics if proven to be effective would be seen as the most significant development in treating psychiatric illness in several decades [3]. 

**Figure 1:**
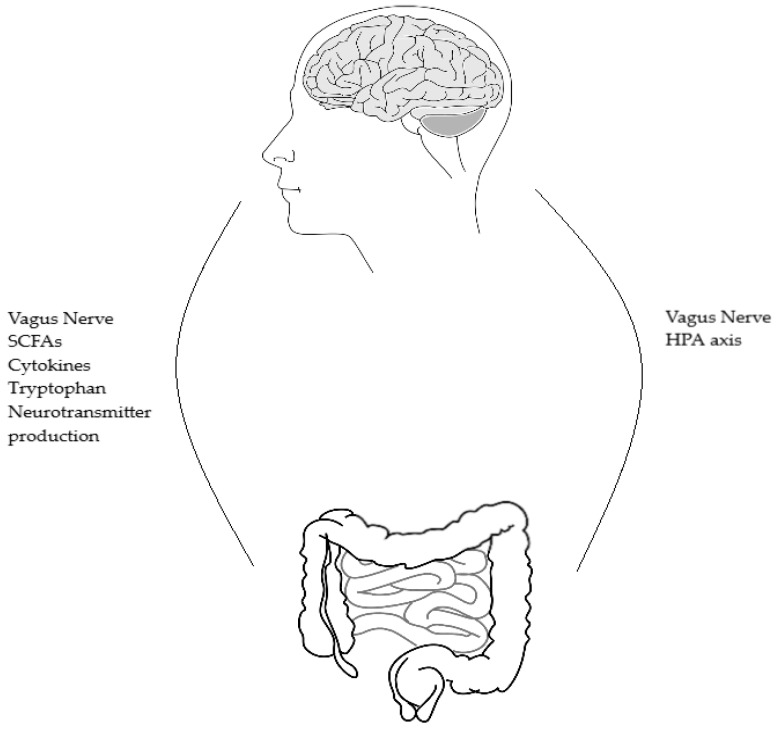
Shows the bidirectional routes of communication between the brain and gut microbes and the reverse, gut microbes and brain. SCFAs = short-chain fatty acids, HPA = hypothalamic-pituitary-adrenal axis.

The microbiome gut brain (MGB) axis is a relatively new concept in the scientific world. The MGB axis has been revealed as a complex communication system which appears to have multiple influences on affect, motivation and higher cognitive functions [4]. Previously, the gut microbiome was not appreciated for the role it plays and the far reach of its influence. In recent years, it has been recognised that the gut microbiota sends messages to the brain via a variety of routes, and from this understanding the MGB axis has emerged as an area of study and even therapeutic promise. The use of germ-free (GF) mice and specific pathogen-free (SPF) mice has allowed research regarding the MGB axis to move forward in a way that was previously not possible. GF mice lack any microorganisms and are microbiologically sterile [5]. SPF mice are free from a specific list of pathogens and are considered to be healthy as test subjects [6]. The bidirectional communication between the gut and the central nervous system plays an important role in maintaining homeostasis [7]. The axis communicates in multiple ways (Figure 1); via the autonomic nervous system, the hypothalamic pituitary adrenal (HPA) axis, the vagus nerve, and the direct production of neurotransmitters and short-chain fatty acids (SCFAs). The multiple functions and routes of communication of the MGB axis and how these relate to each other are not yet clear. Ongoing research is hoped to result in therapeutic solutions to unsolved disease processes in many areas of medicine including psychiatry. The MGB axis links the emotional and cognitive centers of the brain with intestinal functions and permeability, immune activation, enteric reflex and endocrine signaling [4]. Alterations in the gut microbiota effect the multiple routes of communication which make up the MGB axis. It is through these changes that the therapeutic potential is thought to lie. 

Several associations have been suggested between the gut microbiota and health status. Loss of diversity in the gut microbiota has been correlated with increased frailty in elderly patients living in long term care [8]. Certain bacteria are thought to contribute to the pathogenesis of bowel disease and their manipulation may hold therapeutic promise [9]. It has been suggested that irritable bowel syndrome (IBS) and inflammatory bowel disease (IBD) could be part of the same inflammatory spectrum but existing at opposite ends [10]. Faecal microbiome transplants (FMTs) from patients with IBS into GF mice have been found to transfer the symptoms of IBS from humans to mice [11]). Stress-induced alterations in gut microbiota have been linked to sympathetic nervous system activation with cortisol affecting gut wall permeability and microbiota composition [12]. It has also been suggested that the makeup of an individual’s microbiome may influence their susceptibility to psychiatric illness [13]. However, it remains unclear if the changes to the microbiome which have been linked to multiple diseases are causal to the disease process or a secondary effect [14]. The full extent of the interplay between the MGB axis and disease states remains undetermined.

Multiple findings have led to the microbiome being identified as a potential target in the treatment of psychiatric illness. The influence of bacteria on behaviour has been explored, with increased anxiety-like behaviour in mice infected with *Campylobacter jejuni* [15]. Differences in the microbiota in a depressed population have been noted when compared to healthy controls [16]. A change in gut microbiota can produce behavioural signs of depression [17]. This was shown via FMTs from depressed patients into rats with a depleted microbiome. Significant differences across multiple tests have been found between GF mice and SPF mice. It has been suggest that these differences represent decreased anxiety and depression-like behaviour in GF mice [18]. Behavioural changes seen in response to changes in the gut microbiota are thought to be mediated by chemicals originating from the microbiota which act directly and indirectly on the central nervous system [19]. The production of neurotransmitters by the gut microbiome can be considered in the context of the monoamine theory of depression. Similarly, the role of the HPA axis and inflammation in the pathogenesis of depression may be mediated by the gut microbiome communicating with the brain via the HPA axis. From the possibility of alterations of the gut microbiota producing improvements in symptoms of disease, the concept of psychobiotics has emerged. A psychobiotic is a live organism that when ingested in adequate amounts produces mental health benefits [20]. Whether probiotics or psychobiotics will replace the use of prescription medication in the futures remains to be seen. 

Initial research into the MGB axis examined rodent models. Responses to stress and certain behaviours provide some limited information applicable to human disease. Psychological sequelae and physiological changes to stress have both been investigated to further understand the role of the gut microbiome and its therapeutic potential. To date, GF and SPF mice trials of infection, probiotic ingestion, and FMTs have been used to research the role of the gut microbiome [13]. FMTs have resulted in the transferral of behavioural phenotypes between patients diagnosed with depression and rats with a depleted microbiome. This highlights the possible role modifying the gut microbiota could play in treating psychiatric disease [14]. The results in human models are currently limited with research ongoing in multiples centres. This review aims to summarise the current understanding of the MGB axis within a psychiatric context, the functioning of the MGB axis, the pre-clinical and clinical results published to date and the direction of ongoing study. 

## 2. The Human Microbiome

The human microbiome has been estimated to consist of over 100 trillion microbes, with the majority of these living in the gut [21]. The majority of the gut microbiota consist of bacteriodetes and firmicutes [22]. The process of gut colonisation starts at birth, and is influenced by many external factors including method of delivery, diet, hygiene and medication [23]. Although each person’s microbiota is different, the abundance of bacterial phylotypes is alike among healthy individuals [4]. The populations of bacteria present in the gut appear to remain constant for some people, while others experience change [24]. Interestingly, both stability and diversity in the gut microbiome are thought to be necessary to maintain health [25]. A functioning gut microbiome will balance proinflammatory and anti-inflammatory responses which contribute to homeostasis [26]. There are multiple examples indicating that environmental factors can affect gut bacteria. Antibiotics alter the gut microbiota, which can result in decreased resistance to colonisation [27]. An individual’s microbiota is thought to represent their genetic and environmental history and even contributes to their risk of disease and potential treatment response [28]. This vulnerability to external factors has led to the gut microbiota becoming a possible target for disease treatment; if negative events/emotions can result in changes to the microbiota could the process be reversed with changes in the microbiota producing positive outcomes? 

## 3. Functioning of the Human Microbiome

The MGB axis communicates through multiple bidirectional routes. These different modes of communication also interact with each other. The gut microbiota directly and indirectly effects the immune system, in turn the immune system effects the HPA axis and the circulating levels of cytokines [29]. Cytokines send signals via the vagus nerve to the brain and act directly on the blood brain barrier [30].

### 3.1. Hypothalamic Pituitary Adrenal (HPA) Axis

The HPA axis is a centrally acting stress response system which is stimulated by physical or psychological stresses. When a stress is detected, corticotrophin releasing hormone (CRH) is released from the hypothalamus, which induces the release of adrenocorticotrophic hormone (ACTH) from the pituitary gland and glucocorticoids from the adrenal cortex. This cascade differs in response to acute or chronic stress. Chronic stress levels can lead to sustained high levels of glucocorticoids and prolonged activation of the sympathetic and parasympathetic nervous systems. Chronic stress also decreases the feedback mechanism usually occurring when cortisol is secreted. The secretion of proinflammatory cytokines increases circulating levels of glucocorticoids. Stress can impact the gut microbiome through activation of the sympathetic nervous system and slowing of the digestive process, as well as altering the gut barrier [31]. The HPA axis has been shown to be chronically hyperactive in monkeys subjected to early life stress [32]. The physiological changes in these monkeys are similar to those seen in patients diagnosed with post-traumatic stress disorder. HPA axis alterations have been implicated in the pathophysiology of depression, these alterations can be reversed through the administration of antidepressants [33]. Significantly increased stress and immune responses were identified in rat pups exposed to early life maternal [34]. The pups also had increased levels of corticosterone and an altered microbiome. These findings suggest stress impacts not only the HPA axis but also the microbiome. The behavioural and humoral changes were noted to indicate that early life stress has a lasting effect which could contribute to the development of a psychiatric illness. 

Brain-derived neurotrophic factor (BDNF) is a protein which contributes to neuroplasticity. *N*-methyl-d-aspartate (NMDA) receptors are a type of glutamate receptor involved in synaptic plasticity and memory. Decreased levels of BDNF and decreased expression of central *N*-methyl-d-aspartate (NMDA) receptors have been found in stressed mice [35]. Gene expression with decreased NMDA receptors in the amygdala and increased BDNF expression in the hippocampus were demonstrated when anxiety-like behaviour was studied in GF and SPF mice [36]. In patients with depression peripheral BDNF levels have been shown to increase through the administration of antidepressants [37]. Higher levels of ACTH and corticosterone with lower levels of BDNF have been found in GF mice exposed to stress [35]. These changes were reversible through monocolonisation by *Bifidobacterium infantis*. Interestingly, this reversal was time limited. These notable results highlight the role the microbiome plays in the development and functioning of the HPA axis. They also suggest that the development of the HPA axis is time sensitive and early life events can have long-term physiological consequences. The gut microbiome plays a clear role in the development of the HPA axis from birth and across the lifespan [38]. *Lactobacillus farciminis* has been used to decrease the stress response in female rats by impacting gut permeability [39]. Both peripheral and central effects were reported with decreased ACTH and corticosterone in plasma and decreased CRF in the hypothalamus. Improvements in anxiety and depression-like behaviours and cognitive functioning have been reported secondary to *Lactobacillus helveticus* NS8 in chronically stressed SPF rats [40]. Lower levels of corticosterone and ACTH were seen along with increased levels of BDNF mRNA. A significant reduction in the diversity of the gut microbiota after exposure to repeated aggression was noted in mice [41]. A significant increase in the genus *Roseburia* and a significant decrease in the genus *Parabacteroides* was found in mice euthanised 15 h after the last exposure to aggression, compared to mice euthanised immediately after. The influence the microbiome has on the brain has been examined through alterations in the microbiome and the subsequent behaviours [42]. It was suggested that these behavioural changes were independent of the autonomic nervous system, neurotransmitters or inflammation. Increased levels of BDNF were noted after colonisation of GF mice with bacteria from SPF mice treated with antibiotics. Multiple links between the HPA axis and the MGB axis have been found including decreased stress levels in rodent models secondary to ingestion of probiotics. Decreased stress levels are also produced by antidepressant administration. Whether probiotics and antidepressants mediate these changes in the same way remains to be seen. It is clear that preclinical studies have connected the HPA axis and the microbiome suggesting probiotics could play a role in the future of clinical medicine. 

### 3.2. Immune System

Microbial ligands have an influence on the immune system, both at times of rest and inflammatory periods. Bacteria influence the gene expression of immune cells, although how long these influences last is currently unknown [43]. Both the innate and the adaptive immune systems are programmed by commensal bacteria [27]. Host-specific microbiota has been shown to be necessary for the development of a healthy immune system [44]. It has been suggested that disease processes might be caused by dysbiosis, rather than the presence of a particular bacterium leading to the development of a disease. Diseases which have been associated with microbial dysbiosis include obesity, inflammatory bowel disease and diabetes mellitus. Changes in the microbiome secondary to alterations in diet, hygiene practices and antibiotic use during the 20th century have a possible role in chronic inflammatory and autoimmune diseases [43]. The complex relationship between the gut microbiota, the gut wall and the lymphoid tissue and the role this relationship plays in disease has led to the gut microbiota being seen as a potential target for treatment [45].

The permeability of the gut wall is an important factor in interactions between bacteria and the rest of the body [46]. Multiple immune barriers exist in the gut wall which minimise the interaction of enteric bacteria and the systemic immune system [47]. The leaky gut theory of depression suggests that the translocation of bacteria through the gut wall triggers an inflammatory response in patients with depression [48]. The immune response is triggered by the presence of lipopolysaccharide (LPS) which induces a Toll-like receptor (TLR) 4 response. LPS is part of the outer membrane of Gram negative bacteria. It has multiple functions including protection against bile salts and lipophilic antibiotics and triggering of the immune response. TLRs are proteins on the surface of immune cells which recognize patterns and initiate responses of both the innate and adaptive immune systems [49]. Depressed patients were found to have significantly larger IgA and IgM responses to four out of six (Gram negative) bacteria when compared to healthy controls [48]. Chronically depressed patients showed significantly larger IgM responses when compared to both patients with acute depression and healthy controls. These responses are thought to affect gut barrier integrity leading to Gram negative bacteria entering the systemic circulation. Centrally, microglia play a fundamental role in regulating immune responses. A reduced immune response has been found in GF mice, with microglial cytokine and chemokine pathways affected. Gut colonisation has been shown to improve microglial functioning [50]. Increases in cytokines have previously been linked to depressive symptoms [51]. Significantly increased levels of interleukin-6 were found in mice repeatedly exposed to aggression. An immune response did not occur when antibiotics were administered prior to the aggression [41]. A decreased immune cell response with decreased production of cytokines was noted after oral administration of *Bifidobacteria infantis* [52]. To date, limited data is available on how probiotics could ameliorate psychiatric symptoms. The decreased or lack of immune response associated with probiotic ingestion does suggest that some of the causative processes leading to psychiatric illness could be reversed through their ingestion. It is clear that many more questions need to be answered before we start to understand the complexity of the immune system, psychiatric illness and the potential role probiotics could play. 

### 3.3. Vagus Nerve

The vagus nerve is the main pathway between the gut and the brain. It allows signals to move from the gut to the brain and in the opposite direction also [29]. Specific probiotics modulate certain brain functions and behaviours, and in many cases these processes are dependent on the vagus nerve [13]. The vagus nerve exerts several anti-inflammatory effects via contact with the HPA axis, the cholinergic anti-inflammatory pathway and the splenic sympathetic anti-inflammatory pathway. The vagus nerve can differentiate between pathogenic and non-pathogenic bacteria, even in the absence of inflammatory processes [53]. The vagus nerve interacts with the gut microbiota. Direct contact occurs though short-chain fatty acids (SCFAs) which activate afferent vagal fibres. TLRs are expressed on afferent vagal fibres which activate the brain. Vagal afferent fibres do not cross the gut wall epithelium, so signals from the microbiota are indirectly delivered by bacteria or their metabolites crossing the gut wall [54]. 

Anxiety-like behaviour and the anxiolytic effect of *Bifidobacterium longum* were both found to be vagally mediated in mice with chemically-induced colitis [55]. Some preliminary data exists that vagal nerve stimulation could be used as an adjunct in the management of treatment refractory depression, post-traumatic stress disorder, and inflammatory bowel disease. Stimulation of the afferent vagal fibres influences monoamines in the brain stem [56]. Psychobiotic bacteria might be used to stimulate the vagus nerve and in so doing bring about an improvement in depressive symptoms.

### 3.4. Bacteria Producing and Secreting Neurotransmitters

Both neurotransmitters and neuromodulators are produced by bacteria. Gamma amino butyric acid (GABA) is a ubiquitous inhibitory neurotransmitter which is involved in many physiological and psychological processes within the central nervous system. It is produced by certain species of *Lactobacillus* and *Bifidobacterium* [57]. Multiple other neurotransmitters are produced by bacteria including: *Escherichia*, *Bacillus* and *Saccharomyces* spp. producing noradrenaline. *Candida*, *Streptococcus*, *Escherichia* and *Enterococcus* spp. producing serotonin. *Bacillus* producing dopamine and *Lactobacillus* producing acetylcholine [29]. It has been suggested that multiple neurochemicals necessary for neuronal functioning are regulated by the gut microbiota [50]. The production of neurotransmitters indirectly modulates neural signaling in the CNS via molecules released by epithelial cells [14]. 

GABA receptor alterations have been implicated in the pathogenesis of both depressive and anxiety disorders. The ingestion of *Lactobacillus rhamnosus* (JB1) has resulted in alterations of central GABA mRNA expression [58]. Ingestion of this probiotic decreased the production of stress-induced corticosterone, as well as decreasing anxiety and depressive-like behaviours in mice. These results were not found in vagotomised mice supporting the role of the vagal nerve pathway in producing these effects. The potential of manipulating GABA production through probiotics could be another area of research in the future. 

### 3.5. Serotonin and Tryptophan Metabolism 

Tryptophan is an essential amino acid used in the production of protein; it is also a precursor of both serotonin and melatonin. The kynurerine pathway is the primary pathway for tryptophan catabolism [59]. Serotonin is a key neurotransmitter at both ends of the gut brain axis, with the gut microbiota controlling tryptophan catabolism along the kynurenine pathway. Serotonin is released from enterochromaffin cells (EC) secondary to vagal nerve stimulation; ingestion of food; and the presence of acid, amino acids, hypo-osmotic or hyperosmotic solutions in the duodenum [60]. Short-chain fatty acids also stimulate the release of serotonin. The enzymes involved in this pathway are modulated by the immune and sympathetic system, similar to the MGB axis [61]. This is a further example of the interaction of the multiple routes of communication that make up the MGB axis. 

Changes in the serotonergic system have long been implicated in the aetiology of depression [62]. Significantly higher rates of kynurenine and the kynurinine:tryptophan ratio have been found in patients diagnosed with IBS compared to healthy controls [63]. Circulating concentrations of tryptophan are influenced by the gut microbiota [25]. GF male mice have been found to have increased levels of serotonin and 5-Hydroxyindoleacetic acid (5-HIAA), its main metabolite [64]. No change in gene expression was found in these mice. Increased levels of tryptophan have been recorded secondary to the oral administration of *Bifidobacteria infantis* in rats [52]. Many antidepressants currently prescribed target serotonin levels to improve psychiatric symptoms [65]. Is there a possibility that these results could be produced via the ingestion of probiotics instead of traditional antidepressants?

### 3.6. Short Chain Fatty Acids

Short-chain fatty acids (SCFAs) are produced by gut bacteria through the digestion of carbohydrates and protein. Examples of SCFAs include butyrate, proprionate and acetate. SCFAs act through G protein coupled receptors. SCFAs have many roles including cell growth and differentiation, transport and metabolism, and provision of energy for the heart, kidneys and the brain. The production and absorption of SCFAs mainly occurs in the proximal large intestine [25]. SCFAs act as signalling molecules which can affect inflammatory responses. Changes in SCFA concentrations are thought to contribute to the development of both immune and metabolic disorders [66]. SCFAs play a in glucose and energy metabolism, rat models gained less weight compared to controls when given SCFAs (propionate and butyrate) as part of their diet [67]. 

Histone decatylase inhibitors affect gene expression through growth arrest, apoptosis, and differentiation [68]. Butyrate is a histone deacetylase inhibitor; it is neuroprotective and also increases neuroplasticity [69]. It influences serotonin release and vagal nerve stimulation [70]. GF mice have been shown to have higher rates of blood brain barrier (BBB) permeability when compared to SPF mice. Colonisation of GF mice with *Clostridium tyrobutyricum* (which produces butyrate) decreased the BBB permeability to the same level as SPF mice [71]. The administration of probiotics to increase butyrate-producing bacteria has resulted in decreased anxiety-like behaviour in rats and lowered psychological stress in humans [72]. Butyrate and the other SCFAs can be promoted through dietary measures as well as probiotics; whether their ingestion will result in therapeutic solutions has yet to be firmly established. 

## 4. Trials Involving Humans

As one might anticipate, at this point there are far more preclinical than clinical studies targeting the MGB axis. However, preliminary human observational and interventional studies have taken place with some promising results reported. Trials involving both healthy populations and specific patient groups have been carried out including patients diagnosed with major depressive disorder, irritable bowel syndrome, autism spectrum disorder, Parkinson’s disease and schizophrenia. The complex pathology behind many psychiatric disorders is not yet fully understood as well as the role MGB axis plays in it. The complexity of the gut microbiome and its interaction with external factors is highlighted through the use of antibiotics in the treatment of functional bowel disease (FBD). Rifaximin has been trialed in the treatment of IBS [73]. Significant improvements in IBS symptoms up to 10 weeks after the antibiotic was discontinued were noted. Using antibiotics in the management of a functional bowel disorder and producing positive results suggests that the interaction between external factors, the gut microbiome and psychiatric illness could be used to a therapeutic advantage. The link between the symptoms of IBS and the microbiome has been previously established with specific organisms contained in probiotics improving the symptoms of IBS [74]. High rates of psychiatric co-morbidity exist in patients diagnosed with IBS with these patients attending both medical and psychiatric services. The treatment of functional disorders remains a challenge for both services. Improvements of symptoms through probiotics would be welcomed by both medical and psychiatric professionals. 

### 4.1. Observational Studies

Significant differences in the gut microbiota between patients with active major depressive disorder (AMDD) and healthy controls were found at phyla, family and genus levels. Differences were also found at phyla, family and genus levels between patients being treated for major depressive disorder (TMDD) and healthy controls. No significant difference in serum inflammatory biomarkers was found between the depressed groups and the healthy controls. BDNF levels in the AMDD and TMDD groups were lower than those in the healthy controls [75]. An underrepresentation of *Bacteroidales* was found in the faecel microbiota of depressed patients when compared to healthy controls [16]. An altered microbiome was also noted in depressed patients compared to healthy controls with a reduction in *Prevotellaceae* [17]. FMTs from patients with AMDD and from healthy controls into GF mice have resulted in increased Actinobacteria and decreased Bacteroidetes in mice with AMDD faecal matter compared to mice with the healthy control faecel matter. Firmicutes was noted to be responsible for discriminating AMDD from healthy controls, although no significant difference was found between the two groups [18]. Significant differences have been found between the global microbiome and specific operational taxonomic units in patients diagnosed with bipolar affective disorder compared to controls [76]. Although the results of various observational studies have been beneficial in beginning to understand the role the microbiome plays in health status, more interventional studies will need to be carried out to further explore the possible role probiotics could play in clinical medicine. 

### 4.2. Interventional Studies

Administration of a probiotic to a healthy female population did not result in any change in gut microbiota [77]. However, one type of T cell (Th17) was significantly reduced when measured after probiotic administration. A significant decrease was seen in interleukin-6 and interleukin-10 in vitro. These results are thought to show a possible role for probiotics in immunoinflammatory diseases such as depression. Positive results were produced after the oral ingestion of a probiotic for 6 months in a group catergorised as stressed and exhausted. Significant increases were seen in concentration, elation and introversion with significant decreases in fatigue, agitation, sensitivity, anxiety and depression [31]. Decreased anxiety levels were also noted in a healthy population after a 12-week trial of probiotics containing *Lactobacillus gasseri* and *Bifidobacterium longum* [78]. Significantly lowered anxiety and depressive scores were noted in the interventional group in the postpartum period when compared to the placebo group after ingestion of *Lactobacillus rhamnosus* HN001 [79]. Some preclinical results have translated to promising clinical findings with the ingestion of *Bifidobacterium longum* 1714 leading to reduced stress levels and improved memory [80].

Some subscales of the Hopkins Symptom Checklist 90 (somatization, depression and anger-hostility) were significantly decreased after ingestion of a probiotic formulation consisting of *Lactobacillus helveticus* R0052 and *Bifidobacterium longum* R0175 [72]. The cortisol output of the probiotic-treated group decreased significantly over time and the control group’s remained static. This result supports the use of probiotics in the treatment of psychiatric disorders where the HPA axis is implicated in the pathophysiology. Significantly lower levels of *Bifidobacterium* have been found in patients with major depressive disorder compared to healthy controls [81]. They also reported that patients who consumed fermented milk less than once a week had significantly lower levels of *Bifidobacterium* compared to those who consumed it more than once a week. Significant improvements in the Beck Depression Inventory scores were found in patients diagnosed with depressive disorder after 8 weeks ingestion of a probiotic containing *Lactobacillus acidophilus*, *Lactobacillus casei*, and *Bifidobacterium bifidum* [82]. A significant improvement in insulin metabolism and decreased oxidative stress was also found. These results suggest that probiotics could improve the physical sequelae that many patients with depressive disorder experience, as well as improving the actual symptoms of the disorder. Similarly, significant improvements in cognitive functioning and certain metabolic measures were seen in patient’s diagnosed with Alzheimer’s Disease after administration of a probiotic containing *Lactobacillus acidophilus*, *Lactobacillus casei*, *Bifidobacterium bifidum*, and *Lactobacillus fermentum* [83]. Probiotic administration has been studied in patients with a diagnosis of schizophrenia, the probiotic contained *Lactobacillus rhamnosus* and *Bifidobacterium animalis* [84]. No change in the symptoms of schizophrenia were noted. Administration of probiotics earlier in the course of the patient’s illness was suggested as a strategy for further study. To date, the number of interventional studies which have been carried out in psychiatric populations are limited, some of the results reported are positive, the exact role probiotics and the MGB axis will play remains to be discovered.

## 5. Discussion

The possibility that the gut microbiota plays a role in the genesis of psychiatric illness and that the microbiome could act as a therapeutic target is a new paradigm in mental health. To date, some promising results have been reported particularly in preclinical trials, with some translation into clinical trials also. The MGB axis remains a relatively new focus in the research world. To date, significant differences between the microbiome of patients with major depressive disorder and bipolar affective disorder compared to healthy controls have been found. Healthy populations have responded positively to probiotic use. Significant improvements in symptoms were seen in patients with depressive disorder and Alzheimer’s disease after probiotic administration. However, links still need to be made between the differences in the microbiome in disease states and the potential improvements in symptoms from probiotic ingestion. It is not yet known which bacteria are implicated in psychiatric disease or how these bacteria are affected by probiotic ingestion. Many different areas in clinical medicine including psychiatry are hopeful that ongoing research will produce tangible results. However, clearly far more clinical studies are required to determine the real validity of this novel approach. Will psychobiotics or other means of modulating the microbiota be the psychiatric treatments of the future? Certainly this approach shows enormous promise and may provide alternatives to the currently available psychotropic medications and psychological therapies. 

## References

[1] Vigo D., Thornicroft G., Atun R. (2016). Estimating the true global burden of mental illness. Lancet Psychiatry.

[2] Ferguson J.M. (2001). SSRI Antidepressant Medications: Adverse Effects and Tolerability. Prim. Care Companion J. Clin. Psychiatry.

[3] Anglin R., Surette M., Moayyedi P., Bercik P. (2015). Lost in Translation: The Gut Microbiota in Psychiatric Illness. Can. J. Psychiatry Rev. Can. Psychiatr..

[4] Carabotti M., Scirocco A., Maselli M.A., Severi C. (2015). The gut-brain axis: Interactions between enteric microbiota, central and enteric nervous systems. Ann. Gastroenterol. Q. Publ. Hell. Soc. Gastroenterol..

[5] Luczynski P., McVey Neufeld K.-A., Oriach C.S., Clarke G., Dinan T.G., Cryan J.F. (2016). Growing up in a Bubble: Using Germ-Free Animals to Assess the Influence of the Gut Microbiota on Brain and Behavior. Int. J. Neuropsychopharmacol..

[6] O’Rourke J., Lee A., McNeill J. (1988). Differences in the gastrointestinal micro biota of specific pathogen free mice: An often unknown variable in biomedical research. Lab. Anim..

[7] Cryan J.F., O’Mahony S.M. (2011). The microbiome-gut-brain axis: From bowel to behavior. Neurogastroenterol. Motil..

[8] Claesson M.J., Jeffery I.B., Conde S., Power S.E., O’Connor E.M., Cusack S., Harris H.M.B., Coakley M., Lakshminarayanan B., O’Sullivan O. (2012). Gut microbiota composition correlates with diet and health in the elderly. Nature.

[9] O’Hara A.M., Shanahan F. (2006). The gut flora as a forgotten organ. EMBO Rep..

[10] Simrén M., Barbara G., Flint H.J., Spiegel B.M.R., Spiller R.C., Vanner S., Verdu E.F., Whorwell P.J., Zoetendal E.G. (2013). Intestinal microbiota in functional bowel disorders: A Rome foundation report. Gut.

[11] Crouzet L., Gaultier E., Del’Homme C., Cartier C., Delmas E., Dapoigny M., Fioramonti J., Bernalier-Donadille A. (2013). The hypersensitivity to colonic distension of IBS patients can be transferred to rats through their fecal microbiota. Neurogastroenterol. Motil..

[12] Macedo D., Filho A., Nádia Soares de Sousa C., Quevedo J., Barichello T., Vitoriano Nobre Júnior H., De Lucena D. (2017). Antidepressants, antimicrobials or both? Gut microbiota dysbiosis in depression and possible implications of the antimicrobial effects of antidepressant drugs for antidepressant effectiveness. J. Affect. Disord..

[13] Cryan J.F., Dinan T.G. (2012). Mind-altering microorganisms: The impact of the gut microbiota on brain and behaviour. Nat. Rev. Neurosci..

[14] Cenit M.C., Sanz Y., Codoñer-Franch P. (2017). Influence of gut microbiota on neuropsychiatric disorders. World J. Gastroenterol..

[15] Goehler L.E., Park S.M., Opitz N., Lyte M., Gaykema R.P. (2008). Campylobacter jejuni infection increases anxiety-like behavior in the holeboard: Possible anatomical substrates for viscerosensory modulation of exploratory behavior. Brain Behav. Immun..

[16] Naseribafrouei A., Hestad K., Avershina E., Sekelja M., Linløkken A., Wilson R., Rudi K. (2014). Correlation between the human fecal microbiota and depression. Neurogastroenterol. Motil..

[17] Kelly J.R., Borre Y., O’Brien C., Patterson E., El Aidy S., Deane J., Kennedy P.J., Beers S., Scott K., Moloney G. (2016). Transferring the blues: Depression-associated gut microbiota induces neurobehavioural changes in the rat. J. Psychiatr. Res..

[18] Zheng P., Zeng B., Zhou C., Liu M., Fang Z., Xu X., Zeng L., Chen J., Fan S., Du X. (2016). Gut microbiome remodeling induces depressive-like behaviors through a pathway mediated by the host’s metabolism. Mol. Psychiatry.

[19] Collins S.M., Surette M., Bercik P. (2012). The interplay between the intestinal microbiota and the brain. Nat. Rev. Microbiol..

[20] Dinan T.G., Stanton C., Cryan J.F. (2013). Psychobiotics: A Novel Class of Psychotropic. Biol. Psychiatry.

[21] Amon P., Sanderson I. (2017). What is the microbiome?. Arch. Dis. Child. Educ. Pract. Ed..

[22] Mariat D., Firmesse O., Levenez F., Guimarăes V., Sokol H., Doré J., Corthier G., Furet J.-P. (2009). The Firmicutes/Bacteroidetes ratio of the human microbiota changes with age. BMC Microbiol..

[23] Grölund M.-M., Lehtonen O.-P., Eerola E., Kero P. (1999). Fecal Microflora in Healthy Infants Born by Different Methods of Delivery: Permanent Changes in Intestinal Flora After Cesarean Delivery. J. Pediatr. Gastroenterol. Nutr..

[24] Holzapfel W.H., Haberer P., Snel J., Schillinger U., Huis in’t Veld J.H. (1998). Overview of gut flora and probiotics. Int. J. Food Microbiol..

[25] Clarke G., Stilling R.M., Kennedy P.J., Stanton C., Cryan J.F., Dinan T.G. (2014). Minireview: Gut Microbiota: The Neglected Endocrine Organ. Mol. Endocrinol..

[26] Burnet P.W.J., Cowen P.J. (2013). Psychobiotics Highlight the Pathways to Happiness. Biol. Psychiatry.

[27] Clemente J.C., Ursell L.K., Parfrey L.W., Knight R. (2012). The Impact of the Gut Microbiota on Human Health: An Integrative View. Cell.

[28] Foster J.A., McVey Neufeld K.-A. (2013). Gut–brain axis: How the microbiome influences anxiety and depression. Trends Neurosci..

[29] Dinan T.G., Stilling R.M., Stanton C., Cryan J.F. (2015). Collective unconscious: How gut microbes shape human behavior. J. Psychiatr. Res..

[30] Sherwin E., Rea K., Dinan T.G., Cryan J. (2016). A gut (microbiome) feeling about the brain. Curr. Opin. Gastroenterol..

[31] Gruenwald J., Graubaum H.-J., Harde A. (2002). Effect of a probiotic multivitamin compound on stress and exhaustion. Adv. Ther..

[32] Coplan J.D., Andrews M.W., Rosenblum L.A., Owens M.J., Friedman S., Gorman J.M., Nemeroff C.B. (1996). Persistent elevations of cerebrospinal fluid concentrations of corticotropin-releasing factor in adult nonhuman primates exposed to early-life stressors: Implications for the pathophysiology of mood and anxiety disorders. Proc. Natl. Acad. Sci. USA.

[33] Barden N. (2004). Implication of the hypothalamic–pituitary–adrenal axis in the physiopathology of depression. J. Psychiatry Neurosci..

[34] O’Mahony S.M., Marchesi J.R., Scully P., Codling C., Ceolho A.-M., Quigley E.M.M., Cryan J.F., Dinan T.G. (2009). Early Life Stress Alters Behavior, Immunity, and Microbiota in Rats: Implications for Irritable Bowel Syndrome and Psychiatric Illnesses. Biol. Psychiatry.

[35] Sudo N., Chida Y., Aiba Y., Sonoda J., Oyama N., Yu X.-N., Kubo C., Koga Y. (2004). Postnatal microbial colonization programs the hypothalamic–pituitary–adrenal system for stress response in mice. J. Physiol..

[36] Neufeld K.M., Kang N., Bienenstock J., Foster J.A. (2010). Reduced anxiety-like behavior and central neurochemical change in germ-free mice. Neurogastroenterol. Motil..

[37] Castrén E., Võikar V., Rantamäki T. (2007). Role of neurotrophic factors in depression. Curr. Opin. Pharmacol..

[38] Allen A.P., Dinan T.G., Clarke G., Cryan J.F. (2017). A psychology of the human brain–gut–microbiome axis. Soc. Personal. Psychol. Compass.

[39] Ait-Belgnaoui A., Durand H., Cartier C., Chaumaz G., Eutamene H., Ferrier L., Houdeau E., Foramonti J., Bueno L., Theodorou V. (2012). Prevention of gut leakiness by a probiotic treatment leads to attenuated HPA response to an acute psychological stress in rats. Psychoneuroendocrinology.

[40] Liang S., Wang T., Hu X., Luo J., Li W., Wu X., Duan Y., Jin F. (2015). Administration of Lactobacillus helveticus NS8 improves behavioral, cognitive, and biochemical aberrations caused by chronic restraint stress. Neuroscience.

[41] Bailey M.T., Dowd S.E., Galley J.D., Hufnagle A.R., Allen R.G., Lyte M. (2011). Exposure to a Social Stressor Alters the Structure of the Intestinal Microbiota: Implications for Stressor-Induced Immunomodulation. Brain Behav. Immun..

[42] Bercik P., Denou E., Collins J., Jackson W., Lu J., Jury J., Deng Y., Blennerhassett P., Macri J. (2011). The Intestinal Microbiota Affect Central Levels of Brain-Derived Neurotropic Factor and Behavior in Mice. Gastroenterology.

[43] Belkaid Y., Hand T. (2014). Role of the Microbiota in Immunity and inflammation. Cell.

[44] Chung H., Pamp S.J., Hill J.A., Surana N.K., Edelman S.M., Troy E.B., Reading N.C., Villablanca E.J., Wang S., Mora J.R. (2012). Gut Immune Maturation Depends on Colonization with a Host-Specific Microbiota. Cell.

[45] Rhee S.H., Pothoulakis C., Mayer E.A. (2009). Principles and clinical implications of the brain–gut–enteric microbiota axis. Nature reviews. Gastroenterol. Hepatol..

[46] Yarandi S.S., Peterson D.A., Treisman G.J., Moran T.H., Pasricha P.J. (2016). Modulatory Effects of Gut Microbiota on the Central Nervous System: How Gut Could Play a Role in Neuropsychiatric Health and Diseases. J. Neurogastroenterol. Motil..

[47] Feng T., Elson C.O. (2011). Adaptive Immunity in the Host-Microbiota Dialogue. Mucosal Immunol..

[48] Maes M., Kubera M., Leunis J.-C., Berk M. (2012). Increased IgA and IgM responses against gut commensals in chronic depression: Further evidence for increased bacterial translocation or leaky gut. J. Affect. Disord..

[49] Beutler B.A. (2009). TLRs and innate immunity. Blood.

[50] Erny D., de Angelis A.L.H., Jaitin D., Wieghofer P., Staszewski O., David E., Keren-Shaul H., Mahlakoiv T., Jakobshagen K., Buch T. (2015). Host microbiota constantly control maturation and function of microglia in the CNS. Nat. Neurosci..

[51] Felger J.C., Lotrich F.E. (2013). Inflammatory Cytokines in Depression: Neurobiological Mechanisms and Therapeutic Implications. Neuroscience.

[52] Desbonnet L., Garrett L., Clarke G., Bienenstock J., Dinan T.G. (2008). The probiotic Bifidobacteria infantis: An assessment of potential antidepressant properties in the rat. J. Psychiatr. Res..

[53] Forsythe P., Bienenstock J., Kunze W.A., Lyte M., Cryan J.F. (2014). Vagal Pathways for Microbiome-Brain-Gut Axis Communication. Microbial Endocrinology: The Microbiota-Gut-Brain Axis in Health and Disease.

[54] Bonaz B., Bazin T., Pellissier S. (2018). The Vagus Nerve at the Interface of the Microbiota-Gut-Brain Axis. Front. Neurosci..

[55] Bercik P., Park A.J., Sinclair D., Khoshdel A., Lu J., Huang X., Deng Y., Blennerhassett P.A., Fahnestock M., Moine D. (2011). The anxiolytic effect of Bifidobacterium longum NCC3001 involves vagal pathways for gut–brain communication. Neurogastroenterol. Motil..

[56] Breit S., Kupferberg A., Rogler G., Hasler G. (2018). Vagus Nerve as Modulator of the Brain–Gut Axis in Psychiatric and Inflammatory Disorders. Front. Psychiatry.

[57] Barrett E., Ross R.P., O’Toole P.W., Fitzgerald G.F., Stanton C. (2012). γ-Aminobutyric acid production by culturable bacteria from the human intestine. J. Appl. Microbiol..

[58] Bravo J.A., Forsythe P., Chew M.V., Escaravage E., Savignac H.M., Dinan T.G., Bienenstock J., Cryan J.F. (2011). Ingestion of Lactobacillus strain regulates emotional behavior and central GABA receptor expression in a mouse via the vagus nerve. Proc. Natl. Acad. Sci. USA.

[59] Davis I., Liu A. (2015). What is the tryptophan kynurenine pathway and why is it important to neurotherapy?. Expert Rev. Neurother..

[60] Manocha M., Khan W.I. (2012). Serotonin and GI Disorders: An Update on Clinical and Experimental Studies. Clin. Transl. Gastroenterol..

[61] O’Mahony S.M., Clarke G., Borre Y.E., Dinan T.G., Cryan J.F. (2015). Serotonin, tryptophan metabolism and the brain-gut-microbiome axis. Behav. Brain Res..

[62] Owens M.J., Nemeroff C.B. (1994). Role of serotonin in the pathophysiology of depression: Focus on the serotonin transporter. Clin. Chem..

[63] Clarke G., Fitzgerald P., Cryan J.F., Cassidy E.M., Quigley E.M., Dinan T.G. (2009). Tryptophan degradation in irritable bowel syndrome: Evidence of indoleamine 2,3-dioxygenase activation in a male cohort. BMC Gastroenterol..

[64] Clarke G., Grenham S., Scully P., Fitzgerald P., Moloney R.D., Shanahan F., Dinan T.G., Cryan J.F. (2012). The microbiome-gut-brain axis during early life regulates the hippocampal serotonergic system in a sex-dependent manner. Mol. Psychiatry.

[65] Stahl S.M. (1998). Mechanism of action of serotonin selective reuptake inhibitors: Serotonin receptors and pathways mediate therapeutic effects and side effects. J. Affect. Disord..

[66] Ohira H., Tsutsui W., Fujioka Y. (2017). Are Short Chain Fatty Acids in Gut Microbiota Defensive Players for Inflammation and Atherosclerosis?. J. Atheroscler. Thromb..

[67] De Vadder F., Kovatcheva-Datchary P., Goncalves D., Vinera J., Zitoun C., Duchampt A., Bäckhed F., Mithieux G. (2014). Microbiota-Generated Metabolites Promote Metabolic Benefits via Gut-Brain Neural Circuits. Cell.

[68] Monneret C. (2005). Histone deacetylase inhibitors. Eur. J. Med. Chem..

[69] Bourassa M.W., Alim I., Bultman S.J., Ratan R.R. (2016). Butyrate, Neuroepigenetics and the Gut Microbiome: Can a High Fiber Diet Improve Brain Health?. Neurosci. Lett..

[70] Stilling R.M., van de Wouw M., Clarke G., Stanton C., Dinan T.G., Cryan J.F. (2016). The neuropharmacology of butyrate: The bread and butter of the microbiota-gut-brain axis?. Neurochem. Int..

[71] Braniste V., Al-Asmakh M., Kowal C., Anuar F., Abbaspour A., Tóth M., Korecka A., Bakocevic N., Ng L.G., Kundu P. (2014). The gut microbiota influences blood-brain barrier permeability in mice. Sci. Transl. Med..

[72] Messaoudi M., Lalonde R., Violle N., Javelot H., Desor D., Nejdi A., Bisson J.-F., Rougeot C., Pichelin M., Cazaubiel M. (2011). Assessment of psychotropic-like properties of a probiotic formulation (*Lactobacillus helveticus* R0052 and *Bifidobacterium longum* R0175) in rats and human subjects. Br. J. Nutr..

[73] Pimentel M., Park S., Mirocha J., Kane S.V., Kong Y. (2006). The effect of a nonabsorbed oral antibiotic (rifaximin) on the symptoms of the irritable bowel syndrome: A randomized trial. Ann. Intern. Med..

[74] Quigley E.M.M., Flourie B. (2006). Probiotics and irritable bowel syndrome: A rationale for their use and an assessment of the evidence to date. Neurogastroenterol. Motil..

[75] Jiang H., Ling Z., Zhang Y., Mao H., Ma Z., Yin Y., Wang W., Tang W., Tan Z., Shi J. (2015). Altered fecal microbiota composition in patients with major depressive disorder. Brain Behav. Immun..

[76] Evans S.J., Bassis C.M., Hein R., Assari S., Flowers S.A., Kelly M.B., Young V.B., Ellingrod V.E., McInnis M.G. (2017). The Gut Microbiome Composition Associates with Bipolar Disorder and Illness Severity. J. Psychiatr. Res..

[77] Singh A., Sarangi A.N., Goel A., Srivastava R., Bhargava R., Gaur P., Aggarwal A., Aggarwal R. (2018). Effect of administration of a probiotic preparation on gut microbiota and immune response in healthy women in India: An open-label, single-arm pilot study. BMC Gastroenterol..

[78] Nishihira J., Kagami-Katsuyama H., Tanaka A., Nishimura M., Kobayashi T., Kawasaki Y. (2014). Elevation of natural killer cell activity and alleviation of mental stress by the consumption of yogurt containing Lactobacillus gasseri SBT2055 and Bifidobacterium longum SBT2928 in a double-blind, placebo-controlled clinical trial. J. Funct. Foods.

[79] Slykerman R., Hood F., Wickens K., Thompson J., Barthow C., Murphy R., Kang J., Rowden J., Stone P., Crane J. (2017). Effect of Lactobacillus rhamnosus HN001 in Pregnancy on Postpartum Symptoms of Depression and Anxiety: A Randomised Double-blind Placebo-controlled Trial. EBioMedicine.

[80] Allen A.P., Hutch W., Borre Y.E., Kennedy P.J., Temko A., Boylan G., Murphy E., Cryan J.F., Dinan T.G., Clarke G. (2016). Bifidobacterium longum 1714 as a translational psychobiotic: Modulation of stress, electrophysiology and neurocognition in healthy volunteers. Transl. Psychiatry.

[81] Aizawa E., Tsuji H., Asahara T., Takahashi T., Teraishi T., Yoshida S., Ota M., Koga N., Hattori K., Kunugi H. (2016). Possible association of Bifidobacterium and Lactobacillus in the gut microbiota of patients with major depressive disorder. J. Affect. Disord..

[82] Akkasheh G., Kashani-Poor Z., Tajabadi-Ebrahimi M., Jafari P., Akbari H., Taghizadeh M., Memarzadeh M.R., Asemi Z., Esmaillzadeh A. (2016). Clinical and metabolic response to probiotic administration in patients with major depressive disorder: A randomized, double-blind, placebo-controlled trial. Nutrition.

[83] Akbari E., Asemi Z., Daneshvar Kakhaki R., Bahmani F., Kouchaki E., Tamtaji O.R., Hamidi G.A., Salami M. (2016). Effect of Probiotic Supplementation on Cognitive Function and Metabolic Status in Alzheimer’s Disease: A Randomized, Double-Blind and Controlled Trial. Front. Aging Neurosci..

[84] Dickerson F.B., Stallings C., Origoni A., Katsafanas E., Savage C.L.G., Schweinfurth L.A.B., Goga J., Khushalani S., Yolken R.H. (2014). Effect of Probiotic Supplementation on Schizophrenia Symptoms and Association with Gastrointestinal Functioning: A Randomized, Placebo-Controlled Trial. Prim. Care Companion CNS Disord..

